# Neural Responses to Mandatory and Voluntary Donation Impact Charitable Giving Decisions: An Event-Related Potentials Study

**DOI:** 10.3389/fpsyg.2021.783825

**Published:** 2021-12-17

**Authors:** Hongjun Zhang, Hao Ding, Yao Lu, Xuejiao Wang, Danfeng Cai

**Affiliations:** ^1^College of Science and Technology, Ningbo University, Ningbo, China; ^2^Institute of Neuromanagement, College of Science & Technology, Ningbo University, Ningbo, China; ^3^M.I.C.E and Tourism Development Research Base of Ningbo City, Ningbo, China; ^4^Business School, Ningbo University, Ningbo, China

**Keywords:** charitable giving, donation context, donation amount, ERP, P2, LPP

## Abstract

The present study aimed to explore the influence of donation amounts on donation decisions in different donation contexts and to reveal the psychological mechanisms. Furthermore, we focused on how to enhance individuals’ intention to donate voluntarily. We designed an experiment on donation decisions, employing event-related potentials (ERPs) to probe the effect of psychological mechanisms on donation decisions by detecting the neural basis of donation decision-making. Based on S-O-R (stimulus-organism-response) theory, we used donation contexts and donation amounts (stimuli) to induce psychological activity in the participants (organism) and then influence individual donation decision behaviors (response). Moreover, we applied psychological reactance (PR) theory to discuss the effect of donation context on decisions and the corresponding psychological process. The behavioral results showed that donation contexts (mandatory vs. voluntary) were significantly related to the donation amounts (i.e., less vs. more money that the charity received than money that the participants donated). At the ERP level, compared with mandatory donation, voluntary donation evoked a larger P2 amplitude when the charity received less money. In addition, a larger mean amplitude of LPP was elicited by voluntary donation compared to mandatory donation. This study provides practical implications for charity organizers to guide people to donate voluntarily.

## Introduction

Charitable donation is an important part of modern civil society and is a necessary supplement to the government in the public sector (Strang and Park, 2016). Voluntary contributions are vital for some programs (such as art, health care, social welfare, and higher education) in modern society (Mayr et al., 2009). However, people’s intention to donate to charity is not high in most countries. For example, voluntary donation accounted for only 0.14% of the GDP in China in 2019 (Charity alliance, 2019). Therefore, it is critical to explore how to increase people’s willingness to donate voluntarily. To solve this problem, it is necessary to investigate individuals’ motivation to make voluntary donations.

In previous studies, scholars have focused on donation decisions under different donation contexts. For example, Harbaugh et al. (2007) probed the motives for charitable donations, in which they divided donation contexts into mandatory giving (in a passive, tax-like manner) and voluntary giving. They found that subjective satisfaction ratings were higher (on average) in voluntary conditions (Harbaugh et al., 2007). Moreover, some studies have used donation amount as a dimension of donation decision to address the multidimensional nature of the donation decision (Fajardo et al., 2018; Paramita et al., 2020), as well as further study of the need for its interaction with donation contexts. For example, Mayr et al. (2009) indicated that donation amount played an important role in people’s decision to give voluntarily. They suggested it was to confirm that under voluntary conditions, as the amount of donations increases, so does the level of activation and intention to donate (Mayr et al., 2009). However, few studies have focused on the psychological process of donation decisions although it plays a very important role in decision-making (Moon et al., 2015; George and Dane, 2016; Gangl et al., 2017; Tao et al., 2020).

The S-O-R (stimulus-organism-response) theory, which is an extensively applied framework to understand human behavior, posits that environmental stimuli impact human cognitive and affective reactions, thereby influencing behavior (Mehrabian and Russell, 1974). According to S-O-R theory, the donation context and amount, which serve as environmental stimuli, may influence individuals’ psychological processes and subsequent decisions. In addition, psychological reactance (PR) may be another theory that fits our current research questions. PR theory is widely used to address certain phenomena of social influence. According to this theory, if individuals feel that any of their free behaviors is eliminated or threatened with elimination (e.g., in a coercive context), the motivational state of psychological reactance will be aroused (Brehm, 1993; Miron and Brehm, 2006). Thus, the mandatory donations in this study may also induce participants’ psychological mechanisms and corresponding decision-making behaviors. Therefore, in the current study, we intended to apply the S-O-R framework and the PR theory to examine and discuss the effect of donation context and amount on decisions and the corresponding psychological process. Specifically, we focus on two donation contexts, mandatory donation and voluntary donation, as well as the interaction with high and low donation amounts.

For the research method, since psychological scales (e.g., emotional valence and arousal scales) and conventional research methods (e.g., questionnaires, interviews) are not always accurate or objective, neuroscientific methods were employed. Event-related potentials (ERPs) are a neuroscientific method using non-invasive technology that has been repeatedly used to gain insights into social decision-making. Its high temporal resolution enables the mental chronometry of decision-making to be understood in detail (Gangl et al., 2017; Jin et al., 2018). As such, the current study employed ERPs to explore the effect of individual psychological mechanisms on donation decisions as the donation amount changes under different donation contexts.

Many researchers have used ERPs to study related cognitive neural mechanisms (Yoder and Decety, 2014; Jin et al., 2020; Xu et al., 2020). These studies have identified two emotion-related ERP components that have been frequently studied in previous decision neuroscience studies, which are closely related to the processing of attention allocation (P2) and emotional arousal (late positive potential, LPP).

Among ERP components, early components refer to those that appear in the first 300 ms after the onset of a stimulus and have been reported to show the initial sensory encoding of the significant emotional stimulus (Junghöfer et al., 2001; Schupp et al., 2007). Existing ERP studies have proposed that P2 is an attention-related component that indicates early rapid automatic activity. It has been shown that negative stimuli can attract more attention resources and elicit greater amplitudes of P2 than positive stimuli (Carretié et al., 2001; Huang and Luo, 2006; Wang et al., 2012). For example, Zhan et al. (2018) found that a larger P2 was elicited when subjects decided whether to help a stranger compared to a friend during moral decision-making. This was confirmed by the positive correlation between P2 amplitudes and subjective unpleasure (Sarlo et al., 2012; Pletti et al., 2015). Regarding donation, Harbaugh et al. (2007) found that increases in the amounts going to the charity increased the likelihood that a voluntary giving was accepted. They also examined subjective satisfaction ratings as a function of payoffs to the subject and charity in voluntary and mandatory conditions. The results showed that subjective satisfaction increased as the charity received more money than they gave away and, satisfaction was higher in the voluntary conditions than in the mandatory conditions (Harbaugh et al., 2007). This finding suggested that individuals tended to prefer when the charity received more money than they expected. Moreover, previous studies have indicated that people allocate more attentional resources to cost-relevant information when conducting charitable donations, which is reflected by P2 (Gasiorowska et al., 2016; Li et al., 2021). In the current study, subjects might compute more deliberately involving personal costs (money they gave away) and benefits (their final monetary benefits allocated to the charity) under voluntary conditions. We predicted that subjects were unsatisfied when the charity received less money than they gave away, which might induce more negative emotion and capture more attention resources. For mandatory conditions, based on PR theory, participants would be motivationally aroused to engage in control-averse behavior to restore freedom when their freedom of choice was restricted (Brehm, 1993). However, recent studies have also found that social coercion may reduce the sense of agency and the neural processing of the outcomes of one’s own actions (Caspar et al., 2016; Caspar et al., 2018; Villa et al., 2021). In our experimental setting, participants were told that the donation would happen whether they chose “acknowledge,” which might reduce their sense of agency and affect their control-aversion. Thus, we suspected that subjects might not allocate more attention resources regardless of how much money the charity received in these conditions. Therefore, in the current study, we predicted that under voluntary donation, charities receiving less money will attract more attention resources and thereby elicit a larger P2 amplitude (positive polarity) compared to the mandatory donation context.

The other component is late positive potential (LPP), which is a late positive-going component mainly located in the centro-parietal regions of the brain (Cuthbert et al., 2000; Schupp et al., 2007). Moreover, Solomon et al. (2012) demonstrated that the emotional effect on LPP reached significance not only in the posterior region but also in the central and anterior regions. A series of studies have shown that LPP is sensitive to emotional stimuli (Schmitz et al., 2012; Solomon et al., 2012). Therefore, researchers take the difference of LPP amplitude as a marker of emotional regulation processing, reflecting the extent to which individuals can adjust the influence brought by emotional stimulus, the magnitude of which reflects emotional regulation ability (Hajcak et al., 2010). For example, Xu et al. (2020) investigated whether human gifting behavior and brain activity are affected by inequity aversion. They found that the participants were more likely to reject an unfair donation proposal and that the LPP elicited by fair offers was more positive than unfair offers (Hu et al., 2014; Xu et al., 2020). A sense of unfairness reduces charitable giving to a third party (Xu et al., 2020). In this experiment, voluntary donation allowed subjects to choose whether to donate by pressing the “accept” and “reject” buttons, which was a “softer” appeal compared to mandatory behavior. However, for mandatory trials, the donation would happen whether they selected “acknowledge.” As mentioned above regarding PR theory, since their freedom was restricted, they might take action to restore it. However, their sense of agency might be reduced in the mandatory context of this study, further influencing control-averse behavior (Caspar et al., 2017; Caspar et al., 2018; Villa et al., 2021). Hence, participants might be more receptive to the process of voluntary contributions and express a higher level of emotional arousal. We hypothesized that voluntary donation accentuates the emotional impact of donation, as reflected in a larger LPP amplitude, compared to the mandatory condition.

As described above, P2 and LPP may reflect different facets of information processing and intention from the perspective of ERP components. Grounding on S-O-R framework and PR theory, we expected that the impact of donation context and amount on donation willingness would be reflected in the processing of attention allocation (P2) and emotional arousal (late positive potential, LPP).

## Materials and Methods

### Participants

The participants of the current experiment consisted of 28 volunteers (13 males, 15 females). They were undergraduate and graduate students from Ningbo University. Their ages ranged from 19 to 24, with a mean age of 21.46 (*SD* = 1.55). All participants were native Chinese speakers without any history of neurological or psychiatric disorder. They were right-handed and had normal or corrected-to-normal vision. All participants gave written informed consent prior to the experiment. The study was conducted in accordance with the Declaration of Helsinki (WMA, 2009). The protocol was approved by the Academy of Neuroeconomics and Neuromanagement at Ningbo University. Data from one male participant were discarded because of excessive artifacts during electroencephalogram (EEG) recordings. Thus, valid data from 27 participants were entered into the final analysis.

### Materials

This money raised in the experiment on donation decisions was given to the China Youth Development Foundation (CYDF), which is a national charity that helps the growth and development of underprivileged youth through funding services, interest expression and social advocacy. CYDF actually asked for donations through the internet.

To manipulate donation, the experiment developed two donation contexts: mandatory donation, which described giving made by individuals under the pressure of human feelings, or tax-like donation made by entrepreneurs, and voluntary donation. To allow a direct comparison of the effect of both conditions, a within-subjects design was used in which all participants were presented with both conditions. There were 50 different stimuli (2 donation contexts × 25 amount combinations), and all the stimuli were repeated four times. Thus, the whole experiment consisted of 200 trials. Half of the trials were mandatory donations, while the other half were voluntary donations.

**FIGURE 1:**
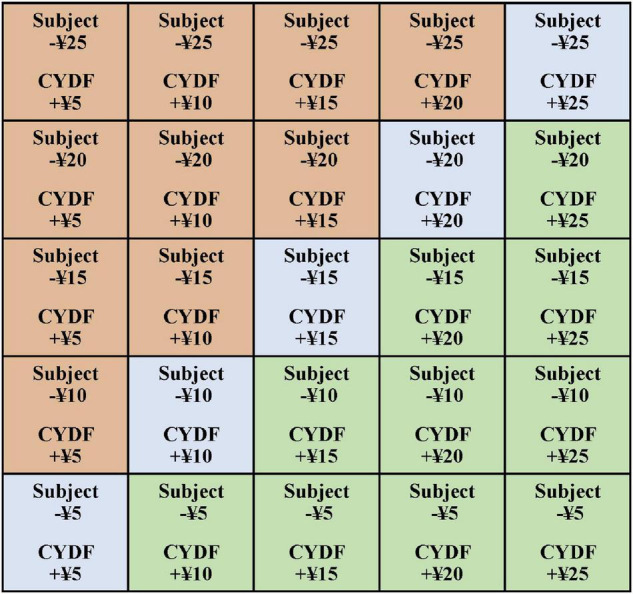
Combinations of the amount of money the participant donated and the amount the charity received.

The amount combinations represented the amount of money that the participant donated and the amount that the charity received, which were 10 combinations of the charity receiving less money than subjects donated, 5 combinations of the charity receiving as much money as the subjects donated, and 10 combinations of the charity receiving more money than the subjects donated (see Figure 1). This experimental design was adapted from the experiment of Harbaugh et al. (2007). According to their experimental design, there were six combinations of less money, four combinations of equal amounts, and six combinations of more money. The main purpose of these manipulations was to provide sufficient variation in the “amount of giving” to elicit a range of individual responses and to reduce participant fatigue (Harbaugh et al., 2007).

After the experiment, subjects were asked to self-report their independence. We designed an independence scale containing seven items, some of which were adapted from the Catell 16 Personality Factor Test (Catell et al., 1970) and Proactive Personality Scale (PPS) (Bateman and Crant, 1993), e.g., “I love being a champion for my ideas, even against others’ opposition,” “I love to plan alone, don’t like interference from others,” and “I do not like to be forced when I do things.” All items were graded on a scale of 1∼5: 1 was strongly disagree, and 5 was strongly agree. We used Cronbach’s α coefficient to test the internal consistency reliability of the scale and applied exploratory factor analysis (EFA) to test the validity of the scale. The results showed that it was reliable and valid (see section “Questionnaire Results”).

### Procedure

Participants were tested individually in a sound-attenuated, shielded chamber, and they were asked to sit 100 cm away from a computer-controlled monitor on which the stimuli were presented. Before the experiment started, participants browsed the material about CYDF’s mission and the experimental instructions for approximately 5 min.

A personal endowment of CNY¥60 was made available for each participant in the ERP experiment, which corresponded to the maximum amount they could obtain for themselves during the experimental task. Participants were told that their decisions on each trial would ultimately affect their final payoff and the monetary benefits allocated to the charity; one mandatory and one voluntary combination would be randomly chosen and implemented after the experiment. They were also told that they were participating in a real donation and that the donation amount would actually be sent through the CYDF website in front of participants. Before the experiment began, the participants were all asked a few questions to ensure that they understood the experiment. They were encouraged to make free choices and were guaranteed anonymity.

**FIGURE 2:**
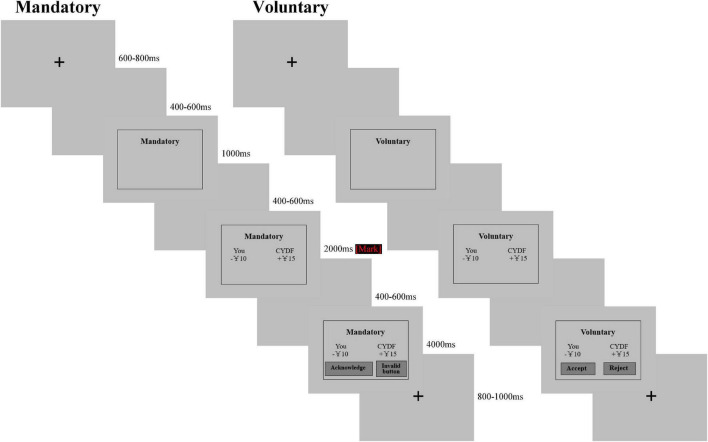
Experimental task: Participants were instructed to make donation decisions in two contexts (mandatory and voluntary). Electroencephalograms (EEGs) were recorded from the subjects throughout the experiment.

Participants were provided with a keypad to report their donation intention for each condition. Events for each trial occurred as presented in the timeline shown in Figure 2. After a 600∼800 ms fixation cross against a gray background, a blank screen lasting for approximately 400∼600 ms followed. Then, the screen revealed whether this trial was mandatory or voluntary for 1,000 ms. After a blank screen appeared for 400∼600 ms, the screen showed how much participants donated and how much CYDF received below mandatory or voluntary for 2,000 ms. Afterward, a blank screen appeared again for 400∼600 ms. Finally, two horizontally arranged labels were added to the lower portion of the screen. For mandatory trials, one of the labels read “acknowledge” (press key “1”) and the other “invalid button” (press key “3”). Participants were told whether they chose to “acknowledge” or not the donation would happen. For voluntary trials, one of the labels read “accept” (press key “1”) and the other “reject” (press key “3”). The participants were asked to make a decision by pressing keys with keypads for 4,000 ms, or the next round would automatically run. Afterward, there was a blank screen with a fixation cross for an intertrial period that was randomly jittered for 800∼1,000 ms (shown in Figure 2). Each subject participated in four 7-min runs of 50 trials. After the experiment, participants completed the self-report questionnaire about independence. Stimuli, recording triggers and response data were presented and recorded using E-Prime 2.0 (Psychology Software Tools, Pittsburgh, PA, United States). The participants were asked to minimize blinks, eye movements, and muscle movements during the whole experiment. The formal experiment started after 6 practice trials.

### Electroencephalogram Recording and Analysis

EEG data were recorded with a cap containing 64 Ag/AgCl electrodes and a Neuroscan Synamp2 Amplifier (Curry8, Neurosoft Labs, Inc.). Its sampling rate was 500 Hz, and channel data were recorded from 0.01 Hz to 100 Hz. The experiment started only when electrode impedances were reduced to below 5 kΩ. A cephalic (forehead) location between FPz and Fz was used as the ground, and the left mastoid served as a reference. To measure eye movements, electrooculograms (EOGs) were recorded from electrodes placed 10 mm from the lateral canthi of both eyes (horizontal EOG) and above and below the left eye (vertical EOG), and EOG artifacts were off-line corrected for all subjects using the method proposed by Semlitsch et al. (1986).

EEG data were off-line transformed based on the average of the left and right mastoid references. EEG recordings were digitally filtered with a low-pass filter at 30 Hz (24 dB/Octave). For ERP analysis, the data were segmented for the epoch from 200 ms before the onset of stimulus on the video monitor to 800 ms after its onset, with the first 200 ms pretarget interval as a baseline. The stimulus was the screen that showed how much participants donated and how much CYDF received below mandatory or voluntary for 2,000 ms. Trials containing amplifier clippings, bursts of electromyography activity, or peak-to-peak deflections exceeding ± 100 μV were excluded. For each participant, EEG recordings were averaged for the four experimental conditions (mandatory-less, mandatory-more, voluntary-less, voluntary-more) over each recording site.

Based on visual observation and the guideline proposed by Picton et al. (2000), we chose the time window of 230∼270 ms for the analysis of P2. Five electrodes (AF3, AF4, F3, Fz, and F4) in the frontal-central area were included in the statistical analysis. A 2 (donation contexts: mandatory vs. voluntary) × 2 (comparison of amount that the charity received: less vs. more) × 5 (electrodes) ANOVA was performed for the P2 analysis. The Bonferroni correction was used for multiple comparisons. We applied Greenhouse-Geisser corrections to determine significance (Greenhouse and Geisser, 1959), and partial eta-squared values (η^2^*_*p*_*) are reported to demonstrate the effect sizes in ANOVA models (Cohen, 1988). Spearman correlation analysis was conducted between the P2 amplitude and participants’ independence of scales in the postquestionnaire.

The time window of 580∼800 ms was chosen from visual inspection of the grand averaged waveforms for the analysis of LPP (Solomon et al., 2012; Hua et al., 2014). We performed the statistical analysis of six electrodes (C3, Cz, C4, CP3, CPz, and CP4). Afterward, a 2 (donation contexts: mandatory vs. voluntary) × 2 (comparison of amount that the charity received: less vs. more) × 6 (electrodes) ANOVA was conducted for the LPP analysis. The Bonferroni correction was used for multiple comparisons. Greenhouse-Geisser corrections were used to determine significance (Greenhouse and Geisser, 1959), and partial eta-squared values (η^2^*_*p*_*) are reported to demonstrate the effect sizes in ANOVA models (Cohen, 1988).

## Results

### Behavioral Results

**FIGURE 3:**
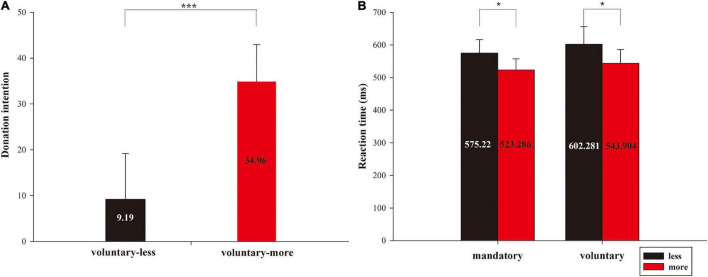
Behavioral results of donation intention and reaction time. **(A)** The donation intentions of the subjects under voluntary conditions. **(B)** The reaction time of the subjects for the four conditions of 2 donation contexts (mandatory vs. voluntary) × 2 amounts (less vs. more). *p < 0.05, ***p < 0.001.

The donation intention of participants and the reaction time between different conditions were analyzed. Participants were told that the donation would happen whether they chose the “acknowledge” or “invalid” button for the mandatory condition; hence, analyzing mandatory donation decisions was unnecessary. However, for the voluntary condition, participants were free to choose “accept” or “reject,” so we measured voluntary donation intention by a frequency of accept/reject responses. Behavioral results are shown in Figure 3A. The pairwise *t*-test was performed for donation intention between comparison of amount that the charity received (less vs. more) under the voluntary condition, and the results showed a significant effect [*t*(1, 26) = –12.947, *p* < 0.001]. This result indicated that the subjects had a higher donation intention when the charity received more money than they donated (*M* = 34.96, S.E. = 8.017) compared to when the charity received less money (*M* = 9.19, S.E. = 10.012) under the voluntary condition.

The reaction time (RT) between different conditions was also analyzed. We conducted a 2 (donation contexts: mandatory vs. voluntary) × 2 (comparison of amount that the charity received: less vs. more) repeated measures ANOVA for the reaction time. As to the multiple comparisons, we performed the Bonferroni correction. Moreover, Greenhouse-Geisser corrections were used to determine significance (Greenhouse and Geisser, 1959), and partial eta-squared values (η^2^*_*p*_*) are reported to demonstrate the effect sizes in ANOVA models (Cohen, 1988). The results showed a significant main effect under different amounts [*F*_(1, 26)_ = 8.107, *p* = 0.008, η^2^*_*p*_* = 0.238], which indicated that the subjects had a longer reaction time when the charity received less money (*M* = 588.251 ms, S.E. = 46.379) compared to more money (*M* = 533.595 ms, S.E. = 35.816) (shown in Figure 3B). However, there was no significant RT difference between the mandatory and voluntary conditions [*F*_(1, 26)_ = 1.002, *p* = 0.326, η^2^*_*p*_* = 0.037], and the interaction effect between donation context and donation amount was also not significant [*F*_(1, 26)_ = 0.058, *p* = 0.812, η^2^*_*p*_* = 0.002].

### Event-Related Potential Results

#### P2 Analysis

**FIGURE 4:**
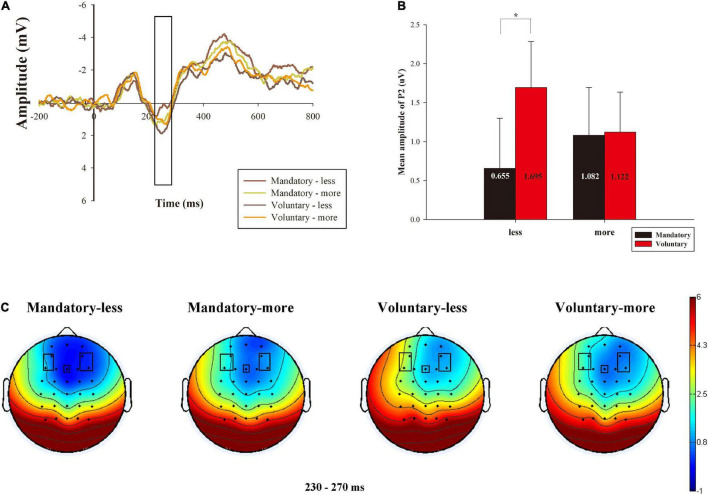
Grand-averaged ERP waveforms of P2 about the average activity of all the electrodes, the mean amplitude of P2 and related brain topography. **(A)** P2 amplitude comparison of the four conditions of 2 donation contexts (mandatory vs. voluntary) × 2 amounts (less vs. more) about the average activity of all the electrodes. **(B)** The mean amplitude of P2 for the four conditions. **(C)** The brain topography of the four conditions and contrast at the P2 time window of 230∼270 ms. *p < 0.05.

As shown in Figure 4A, we conducted a 2 (donation contexts: mandatory vs. voluntary) × 2 (comparison of amount that the charity received: less vs. more) × 5 (electrodes) repeated measures ANOVA for P2 amplitude. The results suggested that donation context significantly interacted with the amount that the charity received [*F*_(1, 26)_ = 7.186, *p* = 0.013, η^2^*_*p*_* = 0.217]. *Post hoc* comparisons with Bonferroni correction indicated that voluntary conditions (*M* = 1.695 μV, S.E. = 0.615) elicited a larger P2 amplitude than mandatory conditions (*M* = 0.655 μV, S.E. = 0.644) when the charity received less money (*p* = 0.015, 95% *CI* of the difference = 0.222–1.860). However, this difference was not significant when the charity received more money (*p* = 0.928). In addition, there was a significant main effect of electrode [*F*_(4, 104)_ = 13.714, *p* = 0.000, η^2^*_*p*_* = 0.345], but we did not find a significant main effect of donation context [*F*_(1, 26)_ = 2.065, *p* = 0.163, η*^2^_*p*_* = 0.074] or the main effect of donation amount [*F*_(1, 26)_ = 0.044, *p* = 0.835, η^2^*_*p*_* = 0.002]. Moreover, the interaction effect between donation context and electrode was not significant [*F*_(4, 104)_ = 2.091, *p* = 0.142, η*^2^_*p*_* = 0.074], neither between donation amount and electrode [*F*_(4, 104)_ = 0.820, *p* = 0.472, η*^2^_*p*_* = 0.031] nor between donation context, donation amount and electrode [*F*_(4, 104)_ = 1.267, *p* = 0.291, η^2^*_*p*_* = 0.046].

We chose the average of all the electrodes (i.e., AF3, AF4, F3, Fz, and F4) and illustrated their neural dynamic activity under different donation contexts and the amount of money received by the charity in Figure 4A. The mean of the four conditions in P2 is displayed in Figure 4B. Meanwhile, the brain topography is shown in Figure 4C, which shows the interactive difference between the four conditions in the frontal-to-central region.

#### Late Positive Potential Analysis

**FIGURE 5:**
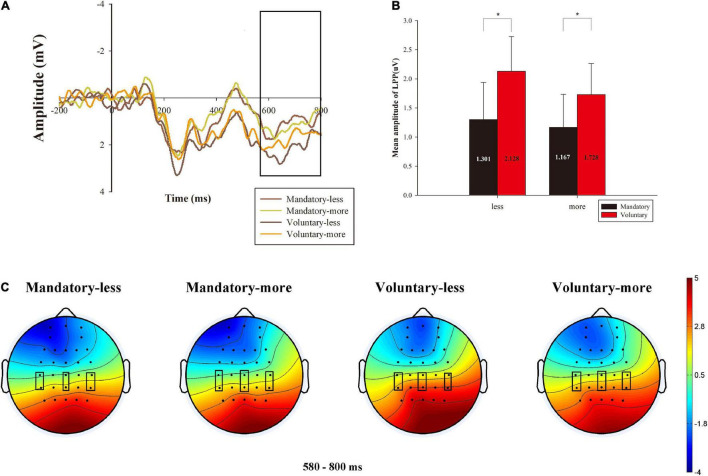
Grand-averaged ERP waveforms of LPP about the average activity of all the electrodes, the mean amplitude of LPP and related brain topography. **(A)** LPP amplitude comparison of the four conditions of 2 donation contexts (mandatory vs. voluntary) × 2 amounts (less vs. more) about the average activity of all the electrodes. **(B)** The mean amplitude of LPP for the four conditions. **(C)** The brain topography of the four conditions and contrast at the LPP time window of 580∼800 ms. *p < 0.05.

A 2 (donation situation: mandatory vs. voluntary) × 2 (comparison of amount that the charity received: less vs. more) × 6 (electrodes) ANOVA for LPP amplitude is shown in Figure 5A, which suggested a significant main effect of donation context [*F*_(1,26)_ = 5.751, *p* = 0.024, η^2^*_*p*_* = 0.181], indicating that a smaller mean amplitude of LPP was elicited under mandatory condition (*M* = 1.234 μV, S.E. = 0.568) compared to voluntary condition (*M* = 1.928 μV, S.E. = 0.497). We also observed a significant main effect of electrode [*F*_(5, 130)_ = 25.826, *p* = 0.000, η^2^*_*p*_* = 0.498]. However, we did not find a significant main effect of different donation amount [*F*_(1, 26)_ = 0.601, *p* = 0.445, η^2^*_*p*_* = 0.023]. In addition, the interaction effect between donation context and donation amount was not significant [*F*_(1, 26)_ = 0.162, *p* = 0.691, η^2^*_*p*_* = 0.006], neither between donation context and electrode [*F*_(5, 130)_ = 0.625, *p* = 0.599, η^2^*_*p*_* = 0.023] nor between donation amount and electrode [*F*_(5, 130)_ = 0.894, *p* = 0.449, η^2^*_*p*_* = 0.033]. There was also no significant interaction effect between donation context, donation amount and electrode [*F*_(5, 130)_ = 0.334, *p* = 0.779, η^2^*_*p*_* = 0.013].

We chose the average of all the electrodes (i.e., C3, Cz, C4, CP3, CPz, and CP4) and illustrated their neural dynamic activity under different donation conditions in Figure 5A. The mean of the four conditions in LPP is displayed in Figure 5B. Meanwhile, the brain topography is shown in Figure 5C, which shows the main difference between the four conditions in the central-to-parietal region.

### Questionnaire Results

We used Cronbach’s α coefficient to test the internal consistency reliability of the independence scale in the questionnaire before analysis. The results showed that the α coefficient was 0.662. Hair et al. (1998) indicated that Cronbach’s α coefficient, which is greater than zero, shows that the scale is more reliable. In exploratory research, the coefficient can be less than 0.7 but should be greater than 0.6. The Cronbach’s α coefficients of independence were greater than 0.6, indicating that the independence scale was reliable. Moreover, we applied EFA to test the validity of the scale. The results showed that the KMO value was 0.628, and Bartlett’s test of sphericity was significant (*p* = 0.000). It has been suggested that only KMO values above 0.60 are acceptable for applying EFA (Kaiser, 1970; Dziuban and Shirkey, 1974), indicating that this independence scale was adequate.

**FIGURE 6:**
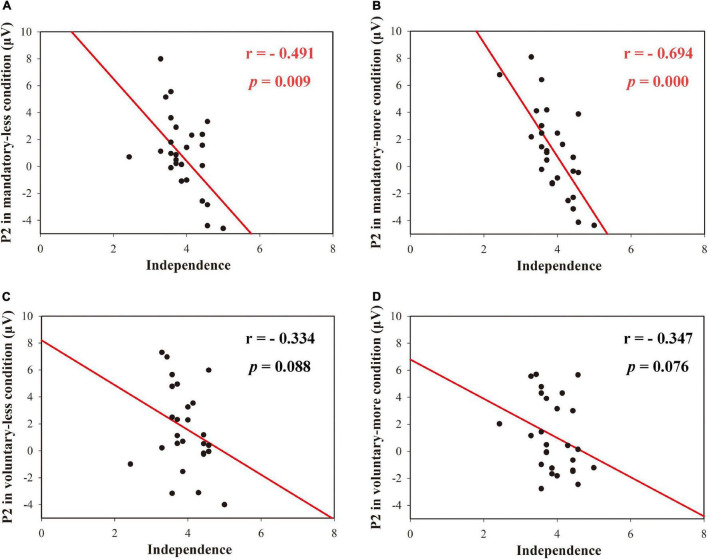
The correlation between amplitudes of P2 in four conditions and independence. Correlation between **(A)** P2 in the mandatory-less condition and independence; **(B)** P2 in the mandatory-more condition and independence; **(C)** P2 in the voluntary-less condition and independence; **(D)** P2 in the voluntary-more condition and independence.

A Spearman correlation analysis between ERP components and independence in the postquestionnaire in four conditions was also conducted. In the mandatory donation condition, there was a significant negative correlation between the mean P2 amplitude and the mean independence when the charity received less or more money (see Figure 6). However, under voluntary conditions, the mean P2 amplitude was not significantly correlated with independence when the charity received less or more money, as shown in Table 1.

**TABLE 1:** Correlation results.

	P2-mandatory		P2-voluntary	
	Less	More	Less	More
Independence	*r* = –0.491** *p* = 0.009	*r* = –0.694** *p* = 0.000	*r* = –0.334 *p* = 0.088	*r* = –0.347 *p* = 0.076

The correlation results amplitudes of P2 in four conditions and independence. **p < 0.01.

## Discussion

Our study emphasized examining whether the donation context and donation amount affect individuals’ donation decisions by detecting the corresponding neural basis. Behaviorally, the donation intention results showed that the subjects in voluntary context had a higher donation intention when the charity received more money compared to when the charity received less money, which supports the findings of previous studies, i.e., when the charity receives more money, people are more likely to donate (Harbaugh et al., 2007). Furthermore, the behavioral result of the reaction time revealed that subjects reacted quicker when the charity received more money than less money, regardless of the mandatory or voluntary donation condition. Previous studies suggested that the task completion time (i.e., reaction time) is positively related to task difficulty and cognitive load (Wang et al., 2015; Jin et al., 2017). Thus, the differential reaction time may indicate that participants required extra cognitive effort when the charity received less money (less level). Thus, when the charity received less money, they might take more time to decide whether to donate. This is consistent with the donation intention results.

Building on the S-O-R theory, we found a significant interaction effect between donation context and donation amount on P2 amplitude at the brain level. The results showed that when the charity received less money, the decision made in the voluntary condition elicited a larger P2 amplitude than that in the mandatory condition. As stated in the introduction, the P2 component is considered to reflect early emotional processes (Zhan et al., 2018). Furthermore, a larger P2 amplitude can reflect automatic mobilization of attention resources to negative stimuli (Carretié et al., 2001; Huang and Luo, 2006; Wang et al., 2012; Jin et al., 2017). Thus, the current findings showed that the charity receiving less money in voluntary donation conditions was a negative stimulus for subjects, leading to greater attention allocation and emotional arousal. This can be explained by the fact that participants paid more attention to the cost-relevant donation information (Ga̧siorowska and Hełka, 2012; Li et al., 2021) and they expected the benefits to equal or exceed the costs in the voluntary conditions. When the charity received more money under voluntary donation conditions, participants were satisfied with the amount and decisively accompanied by weaker negative emotional experiences (Sarlo et al., 2012; Pletti et al., 2015). However, when the charity received less money than they gave away, they were reluctant to accept the amount due to beyond expected outcomes, arousing a stronger negative emotional experience (Zhan et al., 2018). Our behavioral results also indicated that the subjects had a lower donation intention when the charity received less money in voluntary contexts. These results were consistent with those reported in Sarlo et al. (2012) and Pletti et al. (2015). More important, existing studies have suggested that emotions are not always beneficial to moral behavior (Panasiti and Ponsi, 2017; Zhan et al., 2018), e.g., anger enhances immoral behavior (Colasante et al., 2016). Our behavioral results indicated that the subjects had a lower donation intention when the charity received less money in voluntary contexts. This may be because they were not satisfied with the amount of money the charity received, which triggered strong emotions, i.e., more unpleasure made participants engage in less altruistic decisions (Sarlo et al., 2012; Zhan et al., 2018). Thus, there is dissonance between moral content and discrete emotions (Cameron et al., 2015). In addition, participants who behave more morally might tend to donate less to charities (Rahwan et al., 2018), e.g., moral identity decreases donations (Lee et al., 2014). For mandatory conditions, the subjects were asked to donate the money whether they chose “acknowledge,” which restricted their freedom of choice. According to PR theory, the subjects would be aroused to engage in control-averse behavior to reinstate the threatened freedom. However, in the mandatory situation of this study, it’s impossible for subjects to restore eliminated freedom, or it’s pointless to engage in control-averse behavior, so they would reduce their sense of agency (Caspar et al., 2016). If the person realizes that it is impossible to restore freedom, reactance motivation would become low (Miron and Brehm, 2006). Thus, the subjects might pay less attention to the outcomes of their donations. In addition, we also observed that the P2 amplitude in mandatory contexts was negatively related to subjects’ independent personality, indicating that the greater the independence, the lower the P2 amplitude, and the weaker negative emotion participants showed. The subjects paid less attention to how much the charity received because they did not like being forced to donate (i.e., mandatory donation), so they did not allocate more attention resources to negative stimuli in mandatory donations.

We also observed that donation decisions under voluntary conditions elicited a larger LPP amplitude than those under mandatory conditions. The LPP component was believed to be linked to several psychological processes, including attention resource allocation (Hajcak et al., 2010) and emotional arousal (Cuthbert et al., 2000). Here, we argue that the larger LPP component elicited by the voluntary conditions reflects higher emotional arousal than mandatory conditions. As people are more inclined to choose freely according to their own will, participants might tend to voluntarily donate money to the charities, which were considered fair and reasonable, expressing higher emotional arousal, as reflected by a larger LPP amplitude. In mandatory situations that reduced the participants’ freedom to choose donations, based on PR theory, the participants might restore their restricted freedom of choice. Our experimental setting only allows subjects to choose the “acknowledge” in the mandatory condition. Thus, the impossibility of achieving the expected goal reduced their sense of agency and made them give up control-averse behavior (Miron and Brehm, 2006; Caspar et al., 2016), reflecting a lower emotional arousal. Owing to the higher emotional arousal, participants had a higher donation intention in voluntary contexts than in mandatory contexts.

Although both P2 and LPP demonstrate sensitivity to emotional stimuli (Olofsson et al., 2008), their cognitive significance are different. The P2 is an attention-related component that reflects early emotional arousal processing (Carretié et al., 2001; Junghöfer et al., 2001; Schupp et al., 2007; Wang et al., 2012). The LPP is a later component that reflects more sustained processing of emotion (Hajcak et al., 2010; Schmitz et al., 2012; Solomon et al., 2012; Dickey et al., 2021). Thus, P2 and LPP reflect the emotional processing in different cognitive stages. The results of the current study showed that in the early stages of emotional processing (P2), donation context significantly interacted with the amount that the charity received, so there were interactions. However, in the late stages of emotional processing (LPP), the results suggested a significant main effect of donation context, and the interaction effect between donation context and donation amount was not significant. ERP research on developmental changes in emotion regulation is still relatively limited, highlighting a critical direction for future research (Dickey et al., 2021).

The findings of the current study have several implications. First, from the perspective of individual psychological mechanisms, we explored the interactive effect of donation amount and donation context on donation decisions. More importantly, we found that a smaller P2 amplitude would be induced in mandatory conditions than in voluntary conditions when the charity received less money, and voluntary donations would elicit a larger LPP amplitude than mandatory donations. This result provides insight into increasing the willingness to donate voluntarily. Second, we applied ERP technology to examine the effect of S-O-R theory, which provided neuropsychological evidence for individuals’ attentional resources and emotions toward donation contexts. It helps researchers better understand the donation decision-making process and reveals the underlying neural and psychological mechanisms (Camerer and Yoon, 2015; Shen et al., 2018). In addition, the current study also has practical implications for charity organizers. Based on our research, the behavioral results showed that the subjects in voluntary conditions had a higher donation intention when the charity received more money. Additionally, ERP results displayed that when the charity received less money, the decision made in the voluntary condition elicited a larger P2 amplitude, which induced negative emotion. This indicated that participants preferred to more money received by charity. Moreover, we also found that voluntary donations (compared with mandatory situations) lead to higher emotional arousal. To sum up, both in terms of behavioral results and neural responses to donations, voluntary donations are preferred and encourage people to donate more money. Therefore, charity organizers should guide people to donate voluntarily and should not take the form of coercion.

There are some limitations that should be acknowledged. First, the current study did not discuss gender differences. Previous behavioral evidence suggests greater price sensitivity to giving in females (Andreoni and Vesterlund, 2001; Andreoni and Miller, 2002). Women are more likely to donate money to charitable organizations than men (Visser and Roelofs, 2011; Willer et al., 2015; Van Rijn et al., 2019). Thus, it would be valuable to measure the charitable behaviors of female participants and compare their neural activity to those of male participants in future studies. Second, it would be interested to explore the potential role of individual differences in modulating behavior/ERPs in the future research. Although a large number of previous studies on prosocial/altruistic behaviors was conducted from a group-level perspective (Wittek and Bekkers, 2015; Kawamura and Kusumi, 2018; Lee et al., 2021), individual differences are still worth exploring, which is helpful to understanding the individual heterogeneity of prosocial/altruistic behaviors. Third, the sample size for the correlation analysis was relatively small. Although the number of subjects in the current study is sufficient (the effect size of a 2 × 2 repeated-measures ANOVA with 27 subjects can be calculated by G*power to be 0.95), a larger sample size may improve the robustness of the current results, which would further validate the present basic findings.

## Conclusion

In summary, by using the ERP approach, the present study provided electrophysiological evidence for the interactive effect of the donation amount and donation context on individuals’ donation decisions and examined the corresponding psychological process under the S-O-R framework and PR theory. We found that the donation context and the money received by the charity interacted with each other to influence the donation decision at the early stage of rapid automatic processing (P2 amplitude). Especially when the charity received less money, more attentional resources were allocated to obtain voluntary donations compared to mandatory donations and resulted in greater emotional conflict (larger P2 amplitude). In the late stages of emotional processing, compared with mandatory donations, with voluntary giving, participants had a better feeling about the donation scenario, and a higher emotional arousal level was obtained (larger LPP amplitude). This study has several implications for researchers and charity organizers to understand individuals’ willingness to donate voluntarily.

## Data Availability Statement

The raw data supporting the conclusions of this article will be made available by the authors, without undue reservation.

## Ethics Statement

The studies involving human participants were reviewed and approved by the Academy of Neuroeconomics and Neuromanagement at Ningbo University. The patients/participants provided their written informed consent to participate in this study. Written informed consent was obtained from the individual(s) for the publication of any potentially identifiable images or data included in this article.

## Author Contributions

HZ and DC conceived the idea. HZ wrote the draft of the manuscript. YL, HD, and XW prepared the experimental stimuli and collected the data. HZ and HD ran the data analysis and wrote the “Results” section. DC supervised the project. All authors made intellectual contributions to this project and gave approval to the final version of the manuscript for submission.

## Conflict of Interest

The authors declare that the research was conducted in the absence of any commercial or financial relationships that could be construed as a potential conflict of interest.

## Publisher’s Note

All claims expressed in this article are solely those of the authors and do not necessarily represent those of their affiliated organizations, or those of the publisher, the editors and the reviewers. Any product that may be evaluated in this article, or claim that may be made by its manufacturer, is not guaranteed or endorsed by the publisher.
